# Supplementary material for: Bayesian refinement of protein structures and ensembles against SAXS data using molecular dynamics
Source: PLoS Comput Biol. 2017 Oct 18;13(10):e1005800. doi: 10.1371/journal.pcbi.1005800 (PMC5662244; doi:10.1371/journal.pcbi.1005800)
Supplement: S1 Archive — (BZ2) [file pcbi.1005800.s011.tar.bz2 › gromacs_4.62_bayesian_saxs_refi/share/html/online/make_edi.html]

make\_edi


| make\_edi Main Table of Contents |

**VERSION 4.6  
Sat 19 Jan 2013**

---

### Description

make\_edi generates an essential dynamics (ED) sampling input file to be used with mdrun
based on eigenvectors of a covariance matrix (g\_covar) or from a
normal modes analysis (g\_nmeig).
ED sampling can be used to manipulate the position along collective coordinates
(eigenvectors) of (biological) macromolecules during a simulation. Particularly,
it may be used to enhance the sampling efficiency of MD simulations by stimulating
the system to explore new regions along these collective coordinates. A number
of different algorithms are implemented to drive the system along the eigenvectors
(-linfix, -linacc, -radfix, -radacc, -radcon),
to keep the position along a certain (set of) coordinate(s) fixed (-linfix),
or to only monitor the projections of the positions onto
these coordinates (-mon).

References:  
A. Amadei, A.B.M. Linssen, B.L. de Groot, D.M.F. van Aalten and
H.J.C. Berendsen; An efficient method for sampling the essential subspace
of proteins., J. Biomol. Struct. Dyn. 13:615-626 (1996)  
B.L. de Groot, A. Amadei, D.M.F. van Aalten and H.J.C. Berendsen;
Towards an exhaustive sampling of the configurational spaces of the
two forms of the peptide hormone guanylin,
J. Biomol. Struct. Dyn. 13 : 741-751 (1996)  
B.L. de Groot, A.Amadei, R.M. Scheek, N.A.J. van Nuland and H.J.C. Berendsen;
An extended sampling of the configurational space of HPr from E. coli
Proteins: Struct. Funct. Gen. 26: 314-322 (1996)

You will be prompted for one or more index groups that correspond to the eigenvectors,
reference structure, target positions, etc.

-mon: monitor projections of the coordinates onto selected eigenvectors.

-linfix: perform fixed-step linear expansion along selected eigenvectors.

-linacc: perform acceptance linear expansion along selected eigenvectors.
(steps in the desired directions will be accepted, others will be rejected).

-radfix: perform fixed-step radius expansion along selected eigenvectors.

-radacc: perform acceptance radius expansion along selected eigenvectors.
(steps in the desired direction will be accepted, others will be rejected).
**Note:** by default the starting MD structure will be taken as origin of the first
expansion cycle for radius expansion. If -ori is specified, you will be able
to read in a structure file that defines an external origin.

-radcon: perform acceptance radius contraction along selected eigenvectors
towards a target structure specified with -tar.

NOTE: each eigenvector can be selected only once.

-outfrq: frequency (in steps) of writing out projections etc. to .xvg file

-slope: minimal slope in acceptance radius expansion. A new expansion
cycle will be started if the spontaneous increase of the radius (in nm/step)
is less than the value specified.

-maxedsteps: maximum number of steps per cycle in radius expansion
before a new cycle is started.

Note on the parallel implementation: since ED sampling is a 'global' thing
(collective coordinates etc.), at least on the 'protein' side, ED sampling
is not very parallel-friendly from an implementation point of view. Because
parallel ED requires some extra communication, expect the performance to be
lower as in a free MD simulation, especially on a large number of nodes and/or
when the ED group contains a lot of atoms.

Please also note that if your ED group contains more than a single protein,
then the .tpr file must contain the correct PBC representation of the ED group.
Take a look on the initial RMSD from the reference structure, which is printed
out at the start of the simulation; if this is much higher than expected, one
of the ED molecules might be shifted by a box vector.

All ED-related output of mdrun (specify with -eo) is written to a .xvg file
as a function of time in intervals of OUTFRQ steps.

**Note** that you can impose multiple ED constraints and flooding potentials in
a single simulation (on different molecules) if several .edi files were concatenated
first. The constraints are applied in the order they appear in the .edi file.
Depending on what was specified in the .edi input file, the output file contains for each ED dataset

\* the RMSD of the fitted molecule to the reference structure (for atoms involved in fitting prior to calculating the ED constraints)  
\* projections of the positions onto selected eigenvectors  

FLOODING:

with -flood, you can specify which eigenvectors are used to compute a flooding potential,
which will lead to extra forces expelling the structure out of the region described
by the covariance matrix. If you switch -restrain the potential is inverted and the structure
is kept in that region.

The origin is normally the average structure stored in the eigvec.trr file.
It can be changed with -ori to an arbitrary position in configuration space.
With -tau, -deltaF0, and -Eflnull you control the flooding behaviour.
Efl is the flooding strength, it is updated according to the rule of adaptive flooding.
Tau is the time constant of adaptive flooding, high τ means slow adaption (i.e. growth).
DeltaF0 is the flooding strength you want to reach after tau ps of simulation.
To use constant Efl set -tau to zero.

-alpha is a fudge parameter to control the width of the flooding potential. A value of 2 has been found
to give good results for most standard cases in flooding of proteins.
α basically accounts for incomplete sampling, if you sampled further the width of the ensemble would
increase, this is mimicked by α > 1.
For restraining, α < 1 can give you smaller width in the restraining potential.

RESTART and FLOODING:
If you want to restart a crashed flooding simulation please find the values deltaF and Efl in
the output file and manually put them into the .edi file under DELTA\_F0 and EFL\_NULL.

### Files

| option | filename | type | description |
| --- | --- | --- | --- |
| **-f** | eigenvec.trr | Input | Full precision trajectory: trr trj cpt |
| **-eig** | eigenval.xvg | Input, Opt. | xvgr/xmgr file |
| **-s** | topol.tpr | Input | Structure+mass(db): tpr tpb tpa gro g96 pdb |
| **-n** | index.ndx | Input, Opt. | Index file |
| **-tar** | target.gro | Input, Opt. | Structure file: gro g96 pdb tpr etc. |
| **-ori** | origin.gro | Input, Opt. | Structure file: gro g96 pdb tpr etc. |
| **-o** | sam.edi | Output | ED sampling input |

### Other options

| option | type | default | description |
| --- | --- | --- | --- |
| **-[no]h** | bool | no | Print help info and quit || **-[no]version** | bool | no | Print version info and quit || **-nice** | int | 0 | Set the nicelevel || **-xvg** | enum | xmgrace | xvg plot formatting: xmgrace, xmgr or none || **-mon** | string |  | Indices of eigenvectors for projections of x (e.g. 1,2-5,9) or 1-100:10 means 1 11 21 31 ... 91 || **-linfix** | string |  | Indices of eigenvectors for fixed increment linear sampling || **-linacc** | string |  | Indices of eigenvectors for acceptance linear sampling || **-radfix** | string |  | Indices of eigenvectors for fixed increment radius expansion || **-radacc** | string |  | Indices of eigenvectors for acceptance radius expansion || **-radcon** | string |  | Indices of eigenvectors for acceptance radius contraction || **-flood** | string |  | Indices of eigenvectors for flooding || **-outfrq** | int | 100 | Freqency (in steps) of writing output in .xvg file || **-slope** | real | 0 | Minimal slope in acceptance radius expansion || **-linstep** | string |  | Stepsizes (nm/step) for fixed increment linear sampling (put in quotes! "1.0 2.3 5.1 -3.1") || **-accdir** | string |  | Directions for acceptance linear sampling - only sign counts! (put in quotes! "-1 +1 -1.1") || **-radstep** | real | 0 | Stepsize (nm/step) for fixed increment radius expansion || **-maxedsteps** | int | 0 | Maximum number of steps per cycle || **-eqsteps** | int | 0 | Number of steps to run without any perturbations || **-deltaF0** | real | 150 | Target destabilization energy for flooding || **-deltaF** | real | 0 | Start deltaF with this parameter - default 0, nonzero values only needed for restart || **-tau** | real | 0.1 | Coupling constant for adaption of flooding strength according to deltaF0, 0 = infinity i.e. constant flooding strength || **-Eflnull** | real | 0 | The starting value of the flooding strength. The flooding strength is updated according to the adaptive flooding scheme. For a constant flooding strength use -tau 0. || **-T** | real | 300 | T is temperature, the value is needed if you want to do flooding || **-alpha** | real | 1 | Scale width of gaussian flooding potential with alpha^2 || **-[no]restrain** | bool | no | Use the flooding potential with inverted sign -> effects as quasiharmonic restraining potential || **-[no]hessian** | bool | no | The eigenvectors and eigenvalues are from a Hessian matrix || **-[no]harmonic** | bool | no | The eigenvalues are interpreted as spring constant || **-constF** | string |  | Constant force flooding: manually set the forces for the eigenvectors selected with -flood (put in quotes! "1.0 2.3 5.1 -3.1"). No other flooding parameters are needed when specifying the forces directly. |

---

http://www.gromacs.org  
gromacs@gromacs.org
